# mHealth low carbohydrate dietary intervention ameliorates glycaemic profile, blood pressure and weight status in people with type 2 diabetes

**DOI:** 10.1038/s44324-025-00053-6

**Published:** 2025-04-08

**Authors:** Despina Kolivas, Liz Fraser, Ronald Schweitzer, Peter Brukner, George Moschonis

**Affiliations:** 1 School of Allied Health, Human Services & Sport, La Trobe University, Bundoora, VIC 3086 Australia; 2 Watson General Practice, 34 Windeyer Street, Watson, ACT 2602 Australia; 3 East Bentleigh Medical Group, 873 Centre Road, Bentleigh East, VIC 3165 Australia; 4 Department of General Practice, School of Public Health and Preventive Medicine, Monash University, Level 5, 553 St Kilda Rd, Melbourne, VIC 3004 Australia; 5 La Trobe Institute for Sustainable Agriculture & Food (LISAF), La Trobe University, Melbourne, VIC 3086 Australia

**Keywords:** Type 2 diabetes, Type 2 diabetes

## Abstract

Low carbohydrate diets (LCD) have shown efficacy in managing clinical outcomes in type 2 diabetes (T2D). Incorporating digital tools into health care provides an adjunct treatment modality, to educate patients and provide clinical support. This study examines the effect of a mobile health (mHealth) LCD application (app) on glycaemic profile, blood pressure and weight status, in people with T2D. The study is an online single-arm, pre-post study that recruited people with T2D from around Australia, referred via registered supporting general practitioners (GPs). The intervention (Defeat Diabetes app) provides education and resources on the use of a LCD for ongoing management of T2D. After 3 months, our cohort of 99 participants (mean age 59 ± 11 years, 55 females) showed reduced dietary carbohydrate intake as a proportion of overall energy (−14%kJ/day, 95% CI: −17 to −11). Improvement in the primary outcome HbA1c (−1.0%, 95% CI: −1.3 to −0.7), was associated with reduction in dietary carbohydrate intake. Systolic blood pressure (SBP) improved (−6 mmHg, 95% CI: −10 to −1), while 21 participants reduced their diabetes medication dose with two participants discontinuing all diabetes medication. These findings demonstrate that people with T2D receiving LCD education and resources through the Defeat Diabetes app for 3 months improved their glycaemic profile and SBP despite decreased overall medication usage in almost one third of the study sample prescribed medication at baseline.

## Introduction

In 2021, diabetes was estimated to affect 529 million people worldwide, with type 2 diabetes (T2D) accounting for over 96 per cent of these cases^1,2^. Poor glycaemic control, as indicated by increased levels of glycosylated hemoglobin (HbA1c), is associated with increased risk of developing T2D complications over time and places an increasing cost and resource burden on health care systems, presenting a growing challenge for public health^3^. However, T2D is largely preventable and for some people with early diagnosis and management, potentially reversable^4^.

Research evidence shows that the prescription of a low carbohydrate diet (LCD) for management of T2D improves glycaemic control, reduces the need for medications, assists with weight loss, and may improve blood lipid concentrations^5–9^. Clinical guidelines and most research methodologies have generally defined a LCD as one in which less than 26% of energy is derived from carbohydrates (or less than 130 grams per day), while a very low carbohydrate ketogenic diet (VLCKD) is a diet with less than 10% of energy derived from carbohydrates (usually between 20 and 50 grams of carbohydrate per day)^10^. In addition, there is evidence indicating that LCD increases the likelihood of “T2D remission”, which is defined as maintaining “HbA1c < 6.5% measured at least 3 months after cessation of glucose-lowering pharmacotherapy,” and of “T2D reversal”, a term used to describe the return of blood glucose levels to below the threshold diagnostic of diabetes, whilst on metformin^11,12^.

LCDs have been effectively utilised for management of T2D within the primary care setting. The US based Virta Health care group implemented a resource intensive LCD approach (i.e., daily remote monitoring of biometrics, personal health coaches for advice, online peer support and physician supervision) in people with T2D and reported a sustained significant improvement in glycaemic control over a 5-year period, observed despite deprescription of diabetes medication^13^. General practitioners such as Dr David Unwin in the UK, Dr Markins Hawkins in New Zealand and Dr Tro Kalayjian in the US advocate and utilise the LCD approach successfully in their practice with intensive patient support and monitoring^14–16^.

The widespread proliferation of digital technology and the use of digital apps provides access to a new model of care that can support a large proportion of the T2D population and reduce the burden on the healthcare system and the economy. A systematic review and meta-analysis of randomised controlled trials has shown that people with T2D who use digital apps had a 0.30% greater reduction in HbA1c when compared to a control group^17^. Similarly, a systematic literature review examining technology-enabled LCD or VLCKD interventions identified five studies that reported significant reductions in mean HbA1c (ranging from −0.4% to −1.3%) and weight loss (ranging from −3.8 kg to −14 kg) for up to two years^18^.

So far the benefits of LCD for T2D in the primary care context have utilised a multidisciplinary approach that can be resource intensive in providing education and efficient support, and almost exclusively used by private health groups such as Virta Health and individual primary care physicians^13–16^. Education of health care professionals in the wider primary health care community remains a barrier to adopting this innovative approach. The study design allows the opportunity for GPs from around Australia who may be interested to learn about LCD management of T2D and assist in a wide scale research audit with additional support available from the research team. To the best of our knowledge, an experimental study design incorporating the education of health care providers on LCD management of T2D, while utilising the existing health care framework for monitoring and data collection, in combination with the use of a LCD app for education and peer support of patients, has not previously been investigated.

The aim of this study was to examine the effect of a LCD approach delivered online via the Defeat Diabetes digital app on glycaemic profile, blood pressure and weight status in people living with T2D, within the context of the Australian health care system. The current study reports on the findings after the first 3 months of intervention.

## Materials and methods

### Study design and participants

A comprehensive study design protocol has previously detailed the methodology^19^. This single arm pre-post study design follows participants over a 12-month period. The results presented in the current paper represent data collected after 3 months of follow up. Participant data was collected over the period from October 2022 to May 2024.

As medical monitoring of participants is required for the duration of the intervention, only participants referred via registered supporting GPs were eligible to participate. The study was advertised via GP networks, and GPs who were interested in providing support, registered with the research team, who provided detailed study information and patient handouts to assist with recruitment. GPs were provided educational resources and important safety information regarding deprescription of medications for management of T2D with a LCD. The primary inclusion criteria were HbA1c ≥ 6.5%, access to a smartphone or PC, and ability to utilise digital apps. Major inclusion/exclusion criteria are outlined elsewhere^19^.

All people deemed eligible to participate provided their informed consent for inclusion before participating in the study. Data collection was facilitated by online Research electronic data capture (REDCap) case report forms (CRFs) sent via email to GPs and participants^20,21^.

Approval to conduct the study was granted by the La Trobe University Human Research Ethics Committee (HREC) (approval no. 22117, 11 July 2022). The trial was registered with the Australian New Zealand Clinical Trials Registry (ANZCTR) on 17/05/2022, with the ACTRN: 12622000710729.

### Study intervention

After informed consent and baseline data were obtained, participants were granted access to the Defeat Diabetes app and instructed to follow the guidelines over the course of the next 12 months.

The Defeat Diabetes app is a subscription-based commercial app for download on a smartphone (Android and Apple iOS) or use in a web browser and provides a guided educational program on carbohydrate reduction and lifestyle interventions to manage T2D (https://www.defeatdiabetes.com.au/)^22^.

On registration confirmation, the participants were sent a series of emails from Defeat Diabetes explaining how to use the app. They are instructed to follow the video lessons in a sequence and use the associated resources that facilitate further understanding of each particular lesson. The Defeat Diabetes app provides low carbohydrate recipes and cooking demonstration videos, meal planning, shopping lists and exercise plans, as well as a comprehensive recommended food list with a rating system to guide food choices. The Defeat Diabetes app also encourages users to participate in a moderate amount of physical activity to assist with more effective glycaemic control.

Additional support is provided to users with the option to join a private Defeat Diabetes Community Facebook group. App news and events such as the live and recorded webinars are disseminated via a weekly email newsletter to users.

### Outcomes

GP monitoring of patients with T2D is subsidised as part of the Australian Government’s universal health insurance scheme, Medicare^23^. The outcomes reported are based on the medical examinations covered under this scheme, and where they are not, they are optional. All blood samples are collected and analysed at a clinical pathology laboratory (as ordered by each GP) and other measurements are taken on site at each GP practice.

### Primary outcome

The primary outcome was the change in HbA1c from data provided by the participants’ GPs via REDCap CRFs. To secure privacy of personal data, GPs were provided the option of submitting password encrypted data files.

### Secondary outcomes

The participants’ GP also provided data related to secondary outcomes including fasting plasma glucose (FPG), systolic and diastolic blood pressure, and anthropometric markers – body weight, body mass index (BMI) and waist circumference (WC). Prescription medication used for both diabetes and hypertension was recorded at baseline and at 3 months. Adverse events reported by supporting GPs or participants during the follow up period were reported to the Principal Investigator and reviewed by the La Trobe University HREC.

### Participant baseline characteristics

Baseline demographic characteristics included age, sex, country of birth, educational history, and coexisting medical conditions, specifically those that may be related to any of the outcome measures. In addition, the time of diagnosis for T2D was recorded, as the recency of diagnosis may be associated with the likelihood of T2D remission^14,24^. The number of persons in the participant’s household was recorded, as this may provide some background into potential barriers or enablers in the success of the intervention.

### Dietary intake and adherence to the intervention

Three-day food records were completed by participants and submitted via the online REDCap data collection submission forms, via email or via text message to the research team. Data from the food records was entered into FoodWorks Professional 10, Brisbane, Queensland, Australia (Version: 10.0.4266)(2020) and a dietary analysis was obtained at baseline and after 3 months^25^. Where a participant was unable to complete a 3-day food record due to reporting burden, the research team provided the option of a 24-h dietary recall over the phone, following a standardised process by a trained nutritionist. The dietary data served to elucidate the level of carbohydrate reduction and adherence to the intervention.

### Impact of physical activity

Physical Activity levels were monitored using the short version of the International Physical Activity Questionnaire (IPAQ)^26^, which records physical activity levels during the last 7 days. The IPAQ was completed by participants in a self-administered format. The classifications for physical activity as reported by the IPAQ include high, moderate and low. A high level of physical activity equates to more than one hour of moderate intensity physical activity per day. A moderate level of physical activity equates to some activity (about half an hour) of at least moderate intensity physical activity on most days. A low level of physical activity implies that the above criteria are not met.

### Statistical analyses

Continuous variables were examined for the normality of their distribution using the Kolmogorov-Smirnov test. Univariate linear regression models were used to assess within group changes in all continuous study outcomes from baseline to the 3-month follow-up. These regression models were adjusted for appropriate covariates, which included sex, age, time since diabetes diagnosis, co-existing medical conditions, and dietary carbohydrate intake reduction. Changes in categorical variables from baseline to the 3-month follow-up were tested using the Chi-square test. The statistical analyses were conducted for the total sample, and after stratification by gender. Additional stratified analyses were conducted based on co-existing medical conditions and the time since diagnosis of T2D. These results are presented in the supplementary material.

All statistical analyses were performed using SPSS statistical software for Windows (Version 28.0, Armonk, NY). All reported *P* values were two-tailed and the level of statistical significance was *P* < 0.05.

## Results

For purposes of recruitment and at 3 month follow up a total of 99 participants provided data as per the study protocol.

**Fig. 1:**
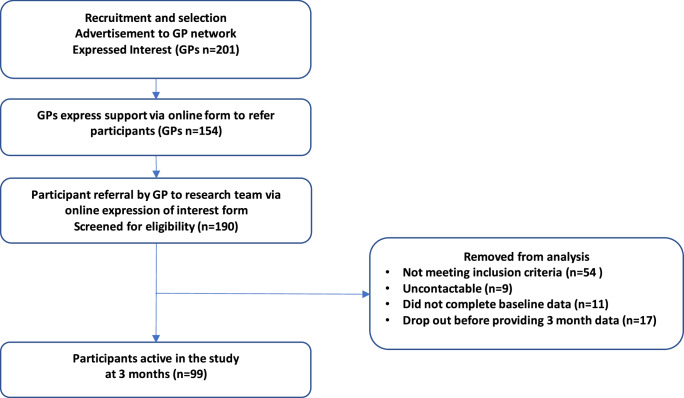
Study process and recruitment.

Figure 1 depicts the study flow from recruitment to 3 months follow up.

### Participant baseline characteristics

Table 1 presents the descriptive characteristics of study participants in terms of the socio-demographics of the total sample (*n* = 99) and by gender. The median duration of diabetes since diagnosis was 2.9 years, with approximately 30% of participants diagnosed within the last year and others having diabetes for more than 20 years. Seventy eight percent of study participants reported having one or more co-existing medical conditions. These included cardiac issues, gastrointestinal disorders, hyperthyroidism, metabolic bone disease, osteoporosis, rheumatoid arthritis, psychological disorders, hypertension, high blood cholesterol, prior gastric bypass surgery, significant kidney or liver disease, and immunodeficiency.

**Table 1:** Descriptive characteristics of study participants at baseline in the total sample and by gender

	Total sample	Male	Female	*P* value
	(*n* = 99)	(*n* = 44)	(*n* = 55)	
Socio-demographics				
Age (years) (Mean (SD))	58.4 (11.3)	57.6 (11.2)	59.1 (11.4)	0.518
Education level (%)				0.636
Up to secondary	34.3	31.8	36.4	
Higher education	65.7	68.2	63.6	
Country of birth (%)				0.129
Australia	60.6	52.3	67.3	
Overseas	39.4	47.7	32.7	
Employment status (%)				0.031
Unemployed	5.1	0	9.3	
Casual/part-time/full-time	63.3	75.0	53.7^a^	
Retired	31.6	25.0	37.0^a^	
No of people in household (%)				0.637
One person	18.2	16.3	20.0	
2 or more people	80.8	83.7	80.0	
Years with type 2 diabetes (median (IQR))	2.9 (6.0)	2.0 (5.1)	4.0 (7.0)	0.017
Time since diagnosis (%)				0.003
Up to 6 years	68.7	79.5	60.0	
6 or more years	31.3	20.5	40.0	
Co-existing medical conditions (%)				0.387
None	22.2	18.2	25.5	
One or more	77.8	81.8	74.5	
Diabetes medications (%)				0.485
No medication	28.3	31.8	25.5	
Medications	71.7	68.2	74.5	
Antihypertensive medications (%)				0.857
No medication	46.5	45.5	47.3	
Medications	53.5	54.5	52.7	
IPAQ activity level (%)				0.495
Low	26.3	20.5	30.9	
Medium	41.4	45.5	38.2	
High	32.3	34.1	30.9	

*P* values that compare continuous variables between genders are derived from the Independent sample T-test or the nonparametric Mann–Whitney test, i.e., as per the normality of their distribution. The *P* values that compare categorical variables are derived from the chi-square test. *p* < 0.05 in the pairwise comparisons in proportions between genders.

^a^Indicates statistically significant pairwise differences between males and females.

### Changes in dietary intake

The changes observed in dietary energy and macronutrient intake from baseline to follow up, are summarised in Table 2. After 3 months of intervention there was a significant decrease in average total energy intake (−1570kJ/day, 95% CI: −2128 to −1013), and this change was observed in both males (−1757kJ/day, 95% CI: −2648 to −867) and females (−1416kJ/day, 95% CI: −2122 to −710).

**Table 2:** Changes in the dietary intake of energy and macronutrients from baseline to 3 months of follow-up in the total sample and by gender

	Baseline			Follow-up			3-Months Change			
Dietary intake of	*n*	Mean	SD	*n*	Mean	SD	Mean change	(95% CI) lower	(95% CI) upper	*P* value
Energy (kJ/day)										
Total sample	99	7969	2378	96	6402	1739	−1570	−2128	−1013	<0.001
Males	44	8791	2357	43	7033	1744	−1757	−2648	−867	<0.001
Females	55	7311	2202	53	5890	1572	−1416	−2122	−710	<0.001
CHO (%kJ/day)										
Total sample	99	32	10	96	18	10	−14	−17	−11	<0.001
Males	44	29	12	43	18	10	−10	−15	−6	<0.001
Females	55	34	8	53	18	10	−17	−20	−13	<0.001
Protein (%kJ/day)										
Total sample	99	22	6	96	27	6	6	4	7	<0.001
Males	44	23	7	43	28	6	5	3	8	<0.001
Females	55	21	4	53	26	6	6	4	7	<0.001
Total fat (%kJ/day)										
Total sample	99	40	7	96	49	10	9	6	11	<0.001
Males	44	41	8	43	46	10	5	2	9	0.007
Females	55	40	7	53	51	9	11	8	14	<0.001
Fibre (g/day)										
Total sample	99	21	8	96	19	9	−2	−5	0	0.074
Males	44	23	9	43	20	10	−3	−7	1	0.185
Females	55	20	7	53	19	8	−2	−4	1	0.237

*P* values were derived from the independent sample T-test and indicate the statistical significance of the changes from baseline to 3 months of follow up.

*CHO* Carbohydrate, *SD* standard deviation from the mean, *CI* confidence interval.

Participants’ macronutrients’ intake expressed as proportion of energy intake changed significantly from baseline to the 3-month follow-up. A significant reduction in dietary carbohydrate intake (−14%kJ/day, 95% CI: −17 to −11) was recorded, while significant increases were observed in protein (6% kJ/day, 95% CI: 4–7) and total fat (9% kJ/day, 95% CI: 6–11) intake. These significant changes were observed in both males and females.

### Changes in physical activity levels

After three months of intervention there were no significant differences in the percentage of participants allocated in the low, medium and high activity physical activity level categories in the total sample and when stratified by gender (data not presented in tables).

### Changes in clinical outcomes

Table 3 summarises the changes observed in the examined clinical outcomes from baseline to follow up. These changes are presented for the total sample, as well as stratified by gender, from baseline to the 3-month follow-up in the examined clinical outcomes. The analyses performed in the total sample showed a significant reduction in HbA1c (−1.0%, 95% CI: −1.3 to −0.7) and FPG (−1.3 mmol/l, 95% CI: −2.1 to −0.6) over 3 months. The changes in HbA1c were more pronounced in males (−1.2%, 95% CI: −1.7 to −0.7) compared to females (−0.8 mmol/l, 95% CI: −1.2 to −0.4). Systolic blood pressure was significantly reduced in the total sample (−6 mmHg, 95% CI: −10 to −1), with no significant differences found between males and females. No significant changes were found in diastolic blood pressure, waist circumference, body weight or BMI in the total sample and by gender. Three participants did not have routine bloodwork at the 3-month follow up and thus their data is missing. A complete subgroup analysis on additional stratification factors other than gender, can be found in the Supplementary Information as Supplementary Table S1.

**Table 3:** Changes in HbA1c, fasting plasma glucose, systolic and diastolic blood pressure, body weight, waist circumference and BMI from baseline to 3 months of follow-up in the total sample and by gender

	Baseline			Follow-up			3-Month Change			
	*n*	Mean	SD	*n*	Mean	SD	Mean change	(95% CI) lower	(95% CI) upper	*P* value
HbA1c %										
Total sample	99	7.7	1.3	96	6.7	0.9	−1.0	−1.3	−0.7	<0.001
Males	44	7.9	1.3	41	6.7	0.9	−1.2	−1.7	−0.7	<0.001
Females	55	7.6	1.3	55	6.8	0.9	−0.8	−1.2	−0.4	<0.001
Fasting plasma glucose mmol/L										
Total sample	93	8.6	2.7	81	7.3	2.2	−1.3	−2.1	−0.6	<0.001
Males	41	8.3	2.2	33	6.9	1.9	−1.4	−2.3	−0.4	0.008
Females	52	8.9	3.1	48	7.6	2.3	−1.4	−2.5	−0.3	0.014
Systolic blood pressure mmHg										
Total sample	99	135	15	92	130	15	−6	−10	−1	0.011
Males	44	138	15	39	132	13	−6	−12	0	0.054
Females	55	133	15	53	128	16	−5	−11	1	0.091
Diastolic blood pressure mmHg										
Total sample	99	83	11	92	80	11	2	−5	1	0.168
Males	44	83	12	39	82	11	−1	−6	4	0.621
Females	55	82	10	53	79	10	−3	−7	1	0.164
Body weight kg										
Total sample	99	98.0	22.2	92	94.0	21.0	−3.9	−10.0	2.1	0.200
Males	44	104.5	20.3	39	100.7	20.0	−3.7	−12.5	5.1	0.406
Females	55	92.7	22.5	53	89.0	20.5	−3.8	−11.8	4.1	0.340
Waist circumference cm										
Total sample	96	113.6	15.3	89	110.6	15.1	−3.0	−7.4	1.4	0.185
Males	42	114.7	15.4	37	112.4	16.4	−2.4	−9.6	4.8	0.506
Females	54	112.8	15.3	52	109.3	14.1	−3.5	−9.1	2.0	0.212
BMI kg/m^2^										
Total sample	99	33.9	6.3	92	32.6	5.9	−1.3	−3.0	0.5	0.150
Males	44	33.3	5.8	39	32.2	5.7	−1.1	−3.6	1.4	0.397
Females	55	34.4	6.7	53	32.9	6.1	−1.5	−3.9	0.9	0.230

*P* values were derived from the independent sample T-test and indicate the statistical significance of the changes from baseline to 3 months of follow up. Statistical analyses were adjusted for age and gender (only in the case of total sample).

*SD* standard deviation from the mean, *CI* confidence interval.

**Fig. 2:**
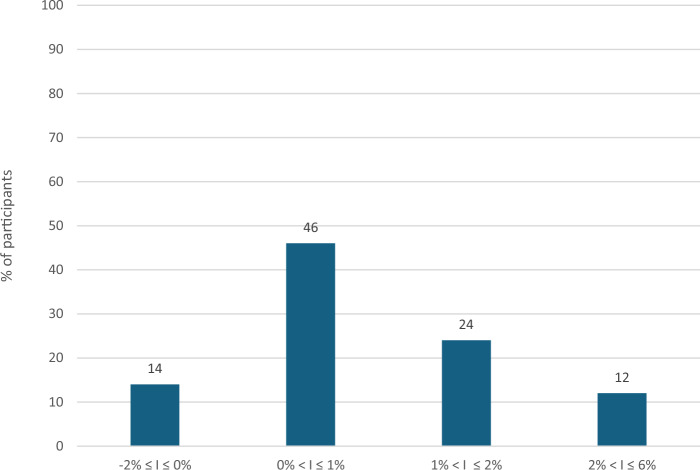
Percentage of participants categorised by reduction in HbA1c level from baseline to the 3-month follow-up. I indicates improvement (reduction) in HbA1c.

Figure 2 shows the percentage of participants categorised by the level of reduction in HbA1c over the 3-month period. Forty six percent of study participants reduced their HbA1c up to 1.0%, 24% of participants between 1% and up to 2%, while 12% of participants by more than 2%. In contrast only 14% of participants experienced either no change or increased their HbA1c over the 3-month period.

**Fig. 3:**
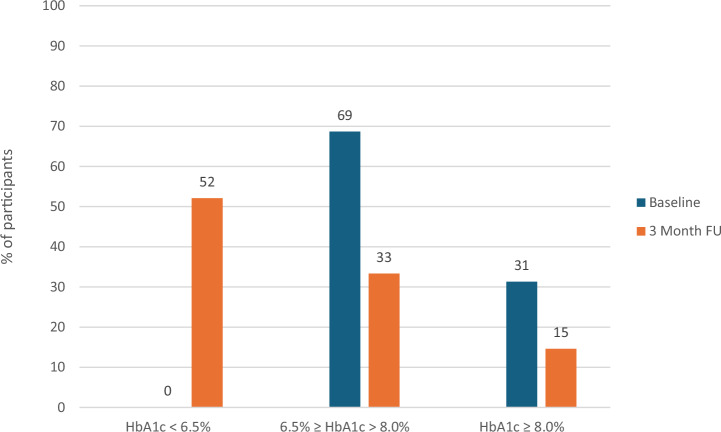
Participants as grouped by HbA1c at baseline and after 3 months of intervention. All participants at baseline had recorded a HbA1c of 6.5% or greater as part of the eligibility criteria.

In addition, Fig. 3 shows that 52% of participants were able to reduce their HbA1c to below the diabetic threshold, defined as achieving a HbA1c of less than 6.5% after 3 months.

Table 4 shows that participants in the total sample who lost more than 5% of their initial body weight over the 3 months had a greater reduction in HbA1c (−0.6%, 95% CI: −1.1 to −0.1) compared to those who did not. This was mainly observed in female participants, while no significant differences were observed in male participants.

**Table 4:** Changes in HbA1c levels by levels of weight loss overall in the total sample and by gender.

	Δ HbA1c (%)						
	≥5% of weight loss			<5% of weight loss (including weight gain)			
	*n*	Mean change	SD	*n*	Mean change	SD	*P* value
Total sample	35	−1.4	1.2	57	−0.8	1.2	0.015
Males	13	−1.8	1.2	26	−1.0	1.6	0.070
Females	22	−1.2	1.1	31	−0.6	0.8	0.046

*P* values that compare continuous variables between genders are derived from the Independent sample T-test or the non parametric Mann–Whitney test, i.e., as per the normality of their distribution. The *p* values that compare categorical variables are derived from the chi-square test. Note: Small sample size is the limitation for gender comparison.

Overall, the majority of participants (89%) lost weight, with 85% recording a decrease in HbA1c after 3 months of the intervention, as shown in Supplementary Table S2.

### Medication use following the intervention

At baseline 28 participants were not prescribed any diabetes medication, while 71 participants were prescribed one or more diabetes medications. After 3 months, 21 participants had reduced their diabetes medication dose with two participants discontinuing all diabetes medication. Six participants had medication added to their management plan, and 42 recorded no change in diabetes medications.

At baseline 53 people were prescribed medication for hypertension, with 46 participants requiring no medication. After 3 months of the intervention 5 participants were able to reduce their medication dose, and 3 people discontinued all hypertension medications. However, two participants had their dose increased and one participant that was not previously on any hypertension medication, had medication added.

### Adherence to the Defeat Diabetes app and its impact on glycaemic control and weight loss

Across the cohort, when comparing those whose average carbohydrate intake was 50 g or less to those with an average carbohydrate intake of greater than 50 g, there was a non-significant decrease in HbA1c (−0.5% 95% CI: −1.0 to 0), together with a significant reduction in body weight (−2.1 kg, 95% CI: −3.4 to −0.7), directly driven by female participants, as shown in Supplementary Table S3 in the Supplementary Information. In particular, participants who reduced their HbA1c, were shown to have also significantly reduced their dietary carbohydrates when grouped by intake of 50 g a day or less, compared to those with a higher dietary intake of carbohydrates (*p* = 0.036).

### Adverse events

Over the 3-month follow up period there were no reported adverse events related to the intervention that required participants to withdraw from the trial. A total of 18 participants sought medical attention throughout the 3-month follow-up period, including six who were admitted to hospital. Reasons for medical attention included cardiac monitoring, work-related stress, impacted bowel, diarrhoea, pain, urinary tract infection and eczema. Five participants sought medical advice for adjustment of medications for diabetes and hypertension. Reasons for hospitalisations included recurrence of atrial fibrillation, infections, colonoscopy and health conditions unrelated to the study intervention.

## Discussion

We conducted a single arm pre-post study that aimed to examine the effect of a digital health intervention on glycaemic control in people with T2D. We found that there was a significant improvement in HbA1c in participants after 3 months of using the Defeat Diabetes app. On average, the amount of dietary carbohydrate consumed by study participants decreased significantly as did overall dietary energy intake. The results of the current study showed that participants compensated for the average reduction in carbohydrate intake by increasing both total fat and protein intake in line with the Defeat Diabetes app recommendations. Increasing levels of protein and fat while lowering the proportion of carbohydrate consumed is likely to result in higher levels of satiety and consequently a reduction in energy intake. While the Defeat Diabetes app provides specific guidance on optimal macronutrient distribution (60% of energy from fat, 30% from protein and 10% from carbohydrates), including specific energy targets for weight maintenance and weight loss, it is left up to the individual to implement the recommendations at a level of adherence to suit their own unique circumstances. With the average age of participants around 58 years, the increased protein intake is likely to be beneficial to overall health given that a higher protein intake is recommended for older adults to protect against sarcopenia^27^. Prior research utilising a self-determined low carbohydrate dietary approach, has shown reductions in dietary fibre over time^28^. This was not the case in our study cohort, where there was no significant change in dietary fibre intake over the 3 month period, indicating that lowering carbohydrate intake did not lead to reductions in dietary fibre intake overall.

There were significant improvements in glycaemic control as evidenced by reduction in HbA1c (−1.0%, 95% CI: −1.3 to −0.7) across the whole study cohort. This is in line with prior research examining the impact of LCD digital interventions (reductions ranging from 0.4% to 1.3%)^18^. This was irrespective of whether participants had a recent diagnosis, longstanding T2D or co-existing medical conditions. As well as the significant improvement in diabetic control, cardiometabolic risk was also improved by a significant reduction in SBP which was recorded across the cohort, specifically for those who had one or more co-existing medical conditions and for those who had a diagnosis of T2D of less than 6 years^29^. These results indicate that the intervention is applicable to a broad range of patient backgrounds, including country of birth, education level and employment status.

There were non statistically significant reductions in body weight recorded, although around one third of the cohort achieved a weight loss of at least 5%. The Royal Australian College of General Practitioners (RACGP) recommends that for optimum management of T2D people should reduce body weight by 5–10%^30^. This advice is consistent with the results seen in the overall sample, with participants achieving a 5% or more of body weight loss having a significantly greater reduction in HbA1c (mean difference −0.6%) compared to those who did not. In addition, in terms of adherence, participants who consumed 50 g of carbohydrate a day or less also had a greater reduction in HbA1c (−0.5%) compared to those who did not. The adherence to a lower level of carbohydrate consumption is also a factor in HbA1c reduction, an association which was found to be statistically significant for female participants in our cohort. The statistical analysis also showed that participants who reduced their HbA1c, had greater adherence to the recommendations in terms of carbohydrate intake, compared to participants that did not. It should be noted that the study cohort had a smaller proportion of male participants and thus a larger effect size was needed to achieve a statistically significant difference in weight loss and adherence, compared to the female study participants. In addition, the female participants in the cohort had double the median “time since diagnosis of T2D” compared to male participants. The longer the time since first diagnosis of T2D, the more difficult it may be to reverse the impacts of T2D, as reported in the DiRECT trial. This may explain why the females in the cohort had lesser reductions in HbA1c when compared to males^31^. It should also be noted that there was no significant change to physical activity levels as determined by the IPAQ questionnaire. This highlights that changes observed in HbA1c were more likely due to the change in dietary intake.

The current dietary guidelines for Australia and New Zealand recommend an acceptable macronutrient distribution range (AMDR) of between 15–25% of energy from protein, 20–35% of energy from fat and 45–65% of energy from carbohydrate for healthy children and adults^32^. An analysis of population data found that there have been significant changes in the macronutrient distribution in Australia over the last 40 years, together with an increase in the levels of obesity^33^. As a proportion of energy, fat intake has reduced and carbohydrate intake has increased, along with an overall increase in energy. Compared to standard dietary advice, a lower proportion of dietary carbohydrate, for people with T2D may be beneficial in achieving better glycaemic control, as demonstrated by the results of the current study. The findings of a recent Australian randomised controlled trial examining the use of a web-based LCD intervention, the T2Diet, in people with T2D, also supports this assertion^34^. The T2Diet intervention focused on food quality and recommended consumption of non starchy vegetables and unprocessed nutrient-dense sources of carbohydrate. The primary dietary recommendation of the T2Diet was to maintain a carbohydrate intake of between 50–100 g per day with no other macronutrient or energy targets. In addition, recommended fat sources were primarily polyunsaturated and monounsaturated, together with reduced fat dairy products^35^. These are in direct contrast to the Defeat Diabetes app, which recommends unprocessed sources of fat, primarily saturated or monounsaturated and full fat dairy products only. Irrespective of these differences, the T2Diet study found over a 4 month period those provided with the web based LCD intervention in combination with standard care, significantly reduced HbA1c by 0.6%, body weight by 3.3 kg, BMI 1.1 kg/m^2^ and the use of diabetes medications, compared to those receiving standard care alone^34^. While the average reduction in HbA1c in the T2Diet study trial (−0.6%) was less than the improvement recorded in the current trial (−1.0%), this provides further support for the use of LCD apps within the context of the Australian health care system.

The results of the current study demonstrate that the use of digital tools to help people with T2D achieve better glycaemic control and improved clinical outcomes, can be achieved with minimal additional health care resources. This is in direct contrast with the DiRECT study in the UK and the DiRECT-Aus trial that seek to achieve T2D remission^31,36^. These interventions were designed to induce weight loss of 10–15 kg or more to generate T2D remission. This was achieved via a very low energy diet (3400–4000 kJ), consisting of a meal replacement formula for up to 12 weeks with gradual food re-introduction. Intensive dietetic support over the course of the intervention (fortnightly for 18 weeks, then monthly), group consultations and ongoing monitoring are primary features. The UK DiRECT study, as well as the DiRECT-Aus trial provide evidence that remission is possible with sustained weight loss maintenance, with 68 out of 138 people, and 86 out of 155 people in the intervention group achieving remission at 12 months in each respective cohort^31,37^. However after 5 years follow up of the UK DiRECT cohort, the number of people able to maintain the weight loss after 5 years is low, with only 12 out of 118 people in the original intervention group remaining in remission^38^. In contrast the Defeat Diabetes app recommends the consumption of whole foods in combination with carbohydrate reduction and achieves improved glycaemic control without the prerequisite of very low energy intake or significant weight loss. A further benefit is that fewer health care resources are utilised to achieve this outcome, especially when compared to the methodology of the DiRECT and DiRECT-Aus trials. The costs of providing meal replacement supplements and ongoing allied health support over a 5-year period, is far greater compared to the cost of providing a digital app. Longer term follow up of participants will provide evidence as to the sustainability of the approach and the likelihood of T2D remission.

Study strengths include the data collection process facilitated via REDCap CRFs for GPs and participants. The research was conducted at relatively low cost as the results obtained from GPs are in alignment with the regular monitoring for T2D as recommended by the RACGP, and was designed to organically fit within the diabetes Annual Cycle of Care^30,39^. Thus the results and applicability of this research have high external validity. Participants and GPs were contacted remotely throughout the study, and participants were recruited from around Australia. The online nature of the study facilitated a wider reach, when compared to a traditional face to face research study. The intervention itself is also relatively low cost when compared to engagement of allied health services, which also may not be conveniently accessible. The peer support feature of the Defeat Diabetes app allows interaction with the broader Defeat Diabetes Facebook Community, and is moderated by health care professionals. This allows users of the app to ask questions and discuss issues, while harnessing the lived experience of the group, providing further timely support.

An additional strength of the current study, was the use of 3 day food records (and as an option 24 h dietary recalls) to get an accurate assessment of dietary intake. Further clarification was sought directly from participants to get a better understanding of the dietary data recorded if required. This allowed participants to complete the food records at a convenient time with minimal reporting burden.

The limitations of the study reflect real world heterogeneity and challenges. Food record data encompassed a variety of different eating patterns (i.e. shift workers, remote site workers, people living alone or with others) also including traditional foods from a range of cultural backgrounds. However, collecting food records has limitations, dietary patterns can change over days or weeks and may not be entirely reflective of the eating behaviour over the entire intervention period. Specifically at baseline with the food record data submitted showing that a small group of participants had decreased their carbohydrate intake after referral but before providing informed consent to participate, due to their eagerness to commence an LCD approach after being notified about the study. Participants were recruited on the understanding that they were able to use digital apps, however there was no way to assess digital competence. In addition, not all participants were Facebook users, and thus would not have had access to peer support.

Referral of participants was exclusively reliant on GPs to provide patient handouts and briefly explain the study details to their patients, who could then register their interest with the research team. Given these circumstances we cannot know how many patients were not willing to adopt a LCD and participate in the intervention. A large number of people referred to the research team by GPs were deemed ineligible, largely due to their HbA1c status being unknown (due to blood test results not being available at the time of referral). Other reasons included already being on a weight loss or LCD, being prescribed insulin or not being able to use digital apps. With respect to data collection, some participants had difficulty with scheduling appointments due to travel, work or the unavailability of their GP. The timing discrepancy reflects the real-world practice of medicine. Some participants did not complete fasting blood tests, and other data such as blood pressure, weight and waist circumference data were not provided to the research team. As mentioned in the protocol paper, we did not provide standardised procedures for the recording of anthropometry, weight data and blood pressure, where results can depend on the time of day taken, and varying technique amongst GPs. We acknowledge these limitations but are more interested in considering the trend over time. This allowed participants to schedule appointments at their convenience and for GPs to work in line with usual T2D monitoring. Another limitation is the lack of a control group. However, the subgroup analysis revealed that participants who had greater adherence to the intervention, including those who had the greatest weight loss, had better overall outcomes and reductions in HbA1c.

Future research will assess the effect of the Defeat Diabetes app over a longer period. Understanding motivation and readiness may inform us as to which participants are more likely to benefit from the intervention^40^. Lifestyle interventions of this nature depend on many factors and adherence may be impacted by indeterminate external factors. In particular, the impact of the intervention could be affected by the underlying motivation and readiness for undertaking lifestyle changes. New features that can be added to the Defeat Diabetes app could include psychological support and motivational aspects for lifestyle changes as these are likely to contribute to adherence, engagement and sustainability. Future studies could compare a case matched cohort utilising conventional medical treatment as a control group for the outcomes measured in study participants. An assessment tool, specifically designed to evaluate LCD quality could also be incorporated in further studies, to better understand overall dietary quality pre- and post intervention and its impact on outcome measures.

We aimed to examine the effect of an LCD app in participants with T2D. We found significant improvements in glycaemic control (i.e., HbA1c and FPG levels), which was associated with reduction in dietary carbohydrates. In addition, there was a significant reduction in systolic blood pressure and a clinically significant reduction in body weight. There was an overall reduction in medication use after 3 months of intervention. The results suggest that the Defeat Diabetes app can provide people with the education, resources and support to help them reduce their carbohydrate intake and HbA1c. Thus it should be considered as an additional tool that health care providers can offer their patients seeking lifestyle modifications to help manage T2D.

## Supplementary information

Supplementary Information

## Data Availability

The data sets used and analysed during this study will be available from the corresponding author G.M. on reasonable request.
