# Supplementary material for: mHealth low carbohydrate dietary intervention ameliorates glycaemic profile, blood pressure and weight status in people with type 2 diabetes
Source: NPJ Metab Health Dis. 2025 Apr 8;3:12. doi: 10.1038/s44324-025-00053-6 (PMC12118682; doi:10.1038/s44324-025-00053-6)
Supplement: Supplementary file 1 — Supplementary Information [file 44324_2025_53_MOESM1_ESM.pdf]

mHealth low carbohydrate dietary intervention ameliorates glycaemic profile, blood pressure and weight status in people with type 2 diabetes

Supplementary Information

Author Information:

Despina Kolivas (ORCID 0000-0003-1195-0688)[1,*], Liz Fraser(ORCID 0009-0000-9108-2440)[2], Ronald Schweitzer(ORCID 0009-0005-2893-7967)[3,4], Peter Brukner(ORCID 0000-0001-9923-4461)[1], and George Moschonis (ORCID 0000-0003-3009-6675)[1,5*]

[1] School of Allied Health, Human Services & Sport, La Trobe University, Bundoora 3086, Australia; d.kolivas@latrobe.edu.au, P.Brukner@latrobe.edu.au, G.Moschonis@latrobe.edu.au

[2] Watson General Practice, 34 Windeyer Street, Watson 2602, Australia.

[3] East Bentleigh Medical Group, 873 Centre Road, Bentleigh East 3165, Australia.

[4] Department of General Practice, School of Public Health and Preventive Medicine, Monash University, Level 5, 553 St Kilda Rd, Melbourne 3004, Australia

[5] La Trobe Institute for Sustainable Agriculture & Food (LISAF), La Trobe University, Melbourne, VIC 3086, Australia

[*] Correspondence: g.moschonis@latrobe.edu.au, d.kolivas@latrobe.edu.au

Table S1 *Changes in HbA1c, fasting plasma glucose, systolic and diastolic blood pressure, body weight, waist circumference and BMI from baseline to 3 months of follow-up in the total sample and by different stratification factors.*

	Baseline			Follow-up			3-months Change			
	**n**	**Mean**	**SD**	**n**	**Mean**	**SD**	**Mean change**	**(95% CI) Lower**	**(95% CI) Upper**	**P value**
**HbA1c %**										
Total Sample	99	7.7	1.3	96	6.7	0.9	-1.0	-1.3	-0.7	<.001
Males	44	7.9	1.3	41	6.7	0.9	-1.2	-1.7	-0.7	<.001
Females	55	7.6	1.3	55	6.8	0.9	-0.8	-1.2	-0.4	<.001
Education Level										
Up to Secondary	34	8.0	1.6	33	6.6	0.7	-1.4	-2.0	-0.8	<.001
Higher education	65	7.6	1.1	63	6.8	1.0	-0.8	-1.1	-0.4	<.001
Country of Birth										
Overseas	39	7.7	1.2	38	6.7	0.8	-1.0	-1.5	-0.5	<.001
Australia	60	7.7	1.4	58	6.8	1.0	-1.0	-1.4	-0.5	<.001
Employment Status										
Unemployed	5	7.3	1.4	5	6.5	1.1	-0.8	-2.2	0.6	0.224
Casual/Part-time/Full-time	62	7.8	1.2	59	6.7	1.0	-1.0	-1.4	-0.6	<.001
Retired	31	7.7	1.5	31	6.8	0.9	-0.9	-1.6	-0.3	0.004
Years with Type 2 Diabetes										
< 6 years	68	7.8	1.4	66	6.7	1.0	-1.0	-1.5	-0.6	<.001
≥ 6 years or more	31	7.6	1.1	30	6.8	0.9	-0.8	-1.3	-0.3	0.003
Coexisting medical conditions										
None	22	7.4	0.9	22	6.6	0.8	-0.9	-1.4	-0.3	0.002
One or more	77	7.8	1.4	74	6.8	1.0	-1.0	-1.4	-0.6	<.001
Activity Level										
Low	26	7.7	1.5	22	6.7	0.9	-1.1	-1.8	-0.4	0.004
Medium	41	7.8	1.3	37	6.9	1.0	-0.8	-1.4	-0.3	0.003
High	32	7.7	1.1	36	6.6	0.9	-1.0	-1.5	-0.5	<.001
Carbohydrate Intake as a %Energy kJ/day (3 months)										
Up to 26% kJ/day	75	7.8	1.4	73	6.7	0.9	-1.1	-1.5	-0.7	<.001
More than 26% kJ/day	21	7.4	1.1	20	6.8	1.0	-0.5	-1.2	0.1	0.095
**Fasting plasma glucose mmol/L**										
Total Sample	93	8.6	2.7	81	7.3	2.2	-1.3	-2.1	-0.6	<.001
Males	41	8.3	2.2	33	6.9	1.9	-1.4	-2.3	-0.4	0.008
Females	52	8.9	3.1	48	7.6	2.3	-1.4	-2.5	-0.3	0.014
Education Level										
Up to Secondary	33	9.0	3.0	26	7.0	1.8	-2.0	-3.3	-0.6	0.005
Higher education	60	8.4	2.6	55	7.4	2.3	-1.0	-1.9	-0.1	0.029
Country of Birth										
Overseas	37	8.9	3.5	33	7.0	1.6	-1.9	-3.2	-0.7	0.003
Australia	56	8.5	2.1	48	7.5	2.5	-1.0	-1.9	-0.1	0.032
Employment Status										
Unemployed	5	8.5	1.8	5	7.4	2.7	-1.1	-3.9	1.6	0.358
Casual/Part-time/Full-time	59	8.6	2.8	52	7.4	2.3	-1.2	-2.2	-0.3	0.012
Retired	28	8.7	2.9	23	7.2	2.0	-1.6	-3.0	-0.1	0.036
Years with Type 2 Diabetes										
< 6 years	64	8.5	2.5	57	7.3	2.3	-1.2	-2.1	-0.3	0.009
≥ 6 years or more	29	8.9	3.3	24	7.2	1.8	-1.8	-3.3	-0.3	0.022
Coexisting medical conditions										
None	22	9.0	3.4	20	6.9	1.5	-2.0	-3.7	-0.3	0.022
One or more	71	8.5	2.5	61	7.4	2.4	-1.1	-2.0	-0.3	0.010
Activity Level										
Low	25	8.5	2.8	18	7.0	2.4	-1.5	-3.2	0.1	0.063
Medium	39	8.8	3.2	33	7.4	2.4	-1.6	-3.0	-0.2	0.029
High	29	8.5	2.0	29	7.3	1.9	-1.1	-2.1	-0.1	0.033
Carbohydrate Intake as a %Energy kJ/day (3 months)										
Up to 26% kJ/day	70	9.1	3.0	61	7.5	2.2	-1.6	-2.5	-0.6	0.001
More than 26% kJ/day	20	7.1	1.2	17	6.6	2.0	-0.6	-1.7	0.5	0.246
**Systolic blood pressure mmHg**										
Total Sample	99	135	15	92	130	15	-6	-10	-1	0.011
Males	44	138	15	39	132	13	-6	-12	0	0.054
Females	55	133	15	53	128	16	-5	-11	1	0.091
Education Level										
Up to Secondary	34	138	18	32	126	14	-12	-20	-4	0.004
Higher education	65	134	14	60	132	15	-2	-7	3	0.343

	Baseline			Follow-up			3-months Change			
	n	Mean	SD	n	Mean	SD	Mean change	(95% CI) Lower	(95% CI) Upper	P value
Country of Birth										
Overseas	39	136	17	37	127	13	-9	-15	-2	0.011
Australia	60	135	14	55	132	16	-4	-9	2	0.206
Employment Status										
Unemployed	5	135	30	5	132	29	-2	-36	32	0.872
Casual/Part-time/Full-time	62	135	14	55	130	14	-6	-11	-1	0.026
Retired	31	135	15	31	130	15	-5	-13	2	0.171
Years with Type 2 Diabetes										
< 6 years	68	138	16	63	131	15	-7	-12	-2	0.011
≥ 6 years or more	31	130	12	29	128	15	-2	-9	4	0.447
Coexisting medical conditions										
None	22	128	11	21	125	10	-3	-10	3	0.299
One or more	77	138	16	71	131	16	-6	-11	-1	0.020
Activity Level										
Low	26	132	10	21	124	11	-8	-14	-1	0.020
Medium	41	139	16	35	133	17	-4	-11	4	0.348
High	32	134	17	35	130	14	-5	-12	2	0.191
Carbohydrate Intake as a %Energy kJ/day (3 months)										
Up to 26% kJ/day	75	136	14	72	130	14	-6	-10	-1	0.010
More than 26% kJ/day	21	134	19	17	133	18	-2	-14	10	0.783
**Diastolic blood pressure mmHg**										
Total Sample	99	83	11	92	80	11	2	-5	1	0.168
Males	44	83	12	39	82	11	-1	-6	4	0.621
Females	55	82	10	53	79	10	-3	-7	1	0.164
Education Level										
Up to Secondary	34	81	13	32	76	10	-4	-10	1	0.123
Higher education	65	83	10	60	82	11	-1	-4	3	0.631
Country of Birth										
Overseas	39	82	13	37	79	11	-2	-8	3	0.383
Australia	60	83	10	55	81	11	-2	-5	2	0.273
Employment Status										
Unemployed	5	85	16	5	80	22	-4	-29	20	0.684
Casual/Part-time/Full-time	62	84	9	55	82	9	-2	-6	1	0.153
Retired	31	78	12	31	77	11	-1	-7	5	0.733
Years with Type 2 Diabetes										
< 6 years	68	85	10	63	83	10	-2	-6	1	0.168
≥ 6 years or more	31	76	11	29	75	10	-2	-7	4	0.550
Coexisting medical conditions										
None	22	82	8	21	79	8	-2	-7	3	0.370
One or more	77	83	11	71	81	12	-2	-5	2	0.297
Activity Level										
Low	26	82	9	21	79	11	-1	-7	4	0.629
Medium	41	83	13	35	81	11	-2	-7	4	0.554
High	32	82	9	35	81	10	-1	-6	4	0.703
Carbohydrate Intake as a %Energy kJ/day (3 months)										
Up to 26% kJ/day	75	82	11	72	81	11	-2	-5	2	0.337
More than 26% kJ/day	21	82	9	17	80	11	-2	-8	4	0.564
**Body weight kg**										
Total Sample	99	98.0	22.2	92	94.0	21.0	-3.9	-10.0	2.1	0.200
Males	44	104.5	20.3	39	100.7	20.0	-3.7	-12.5	5.1	0.406
Females	55	92.7	22.5	53	89.0	20.5	-3.8	-11.8	4.1	0.340
Education Level										
Up to Secondary	34	97.0	21.9	32	94.1	19.4	-3.0	-12.2	6.1	0.513
Higher education	65	98.4	22.6	60	94.0	22.0	-4.2	-11.8	3.5	0.284
Country of Birth										
Overseas	39	93.2	20.5	37	89.6	20.2	-3.3	-11.5	5.0	0.431
Australia	60	101.1	22.9	55	96.9	21.2	-4.1	-10.9	2.7	0.237
Employment Status										
Unemployed	5	109.2	33.0	5	106.0	28.5	-3.2	-50.3	44.0	0.879
Casual/Part-time/Full-time	62	100.3	21.7	56	95.8	20.9	-4.3	-12.0	3.5	0.278
Retired	31	92.0	21.0	30	89.0	19.5	-3.6	-11.7	4.5	0.376
Years with Type 2 Diabetes										
< 6 years	68	101.4	21.4	64	97.3	19.6	-4.1	-10.8	2.5	0.223
≥ 6 years or more	31	90.5	22.5	28	86.5	22.4	-3.5	-15.1	8.2	0.554
Coexisting medical conditions										
None	22	93.0	19.0	21	88.0	17.8	-5.2	-16.4	6.1	0.359

	Baseline			Follow-up			3-months Change			
	n	Mean	SD	n	Mean	SD	Mean change	(95% CI) Lower	(95% CI) Upper	P value
One or more	77	99.4	23.0	71	95.8	21.6	-3.2	-9.7	3.4	0.342
Activity Level										
Low	26	100.6	18.2	21	92.5	19.8	-9.5	-20.4	1.3	0.084
Medium	41	101.2	24.4	36	94.5	22.2	-6.4	-16.6	3.8	0.216
High	32	91.7	21.7	34	95.0	21.0	2.3	-7.7	12.3	0.647
Carbohydrate Intake as a %Energy kJ/day (3 months)										
Up to 26% kJ/day	75	100.1	22.1	72	95.9	21.7	-3.8	-10.5	2.9	0.259
More than 26% kJ/day	21	89.4	20.8	18	86.2	17.2	-3.0	-14.7	8.7	0.609
**Waist Circumference cm**										
Total Sample	96	113.6	15.3	89	110.6	15.1	-3.0	-7.4	1.4	0.185
Males	42	114.7	15.4	37	112.4	16.4	-2.4	-9.6	4.8	0.506
Females	54	112.8	15.3	52	109.3	14.1	-3.5	-9.1	2.0	0.212
Education Level										
Up to Secondary	34	114.6	14.2	32	111.1	14.7	-3.0	-9.9	3.8	0.382
Higher education	62	113.1	16.0	57	110.4	15.4	-2.8	-8.5	3.0	0.345
Country of Birth										
Overseas	37	110.8	14.9	33	108.2	15.3	-2.9	-9.5	3.8	0.391
Australia	59	115.4	15.5	56	112.0	14.9	-3.1	-8.2	2.0	0.230
Employment Status										
Unemployed	5	123.1	20.8	5	119.2	17.5	-3.9	-33.2	25.4	0.762
Casual/Part-time/Full-time	60	113.3	15.1	53	110.6	14.9	-2.8	-8.4	2.8	0.327
Retired	30	112.5	15.2	30	108.9	15.3	-4.3	-11.1	2.5	0.215
Years with Type 2 Diabetes										
< 6 years	66	115.0	14.9	62	111.3	14.7	-3.7	-8.8	1.5	0.164
≥ 6 years or more	30	110.7	16.2	27	109.1	16.0	-1.4	-10.2	7.3	0.741
Coexisting medical conditions										
None	21	112.4	14.4	19	106.5	12.4	-6.3	-14.6	1.9	0.130
One or more	75	114.0	15.7	70	111.7	15.6	-2.1	-7.1	2.9	0.412
Activity Level										
Low	25	116.0	13.0	21	111.1	13.0	-6.8	-14.5	0.9	0.081
Medium	41	115.8	17.8	34	111.8	15.7	-4.3	-12.1	3.6	0.282
High	30	108.7	12.5	33	109.6	16.0	0.7	-6.7	8.0	0.855
Carbohydrate Intake as a %Energy kJ/day (3 months)										
Up to 26% kJ/day	73	115.7	15.7	68	112.4	15.5	-3.3	-8.5	1.9	0.215
More than 26% kJ/day	20	106.0	10.6	18	103.6	11.5	-2.2	-9.4	5.1	0.546
**BMI kg/m^2**										
Total Sample	99	33.9	6.3	92	32.6	5.9	-1.3	-3.0	0.5	0.150
Males	44	33.3	5.8	39	32.2	5.7	-1.1	-3.6	1.4	0.397
Females	55	34.4	6.7	53	32.9	6.1	-1.5	-3.9	0.9	0.230
Education Level										
Up to Secondary	34	33.8	6.4	32	32.7	5.7	-1.1	-4.0	1.8	0.453
Higher education	65	34.0	6.3	60	32.6	6.0	-1.4	-3.6	0.8	0.206
Country of Birth										
Overseas	39	32.6	5.8	37	31.5	5.4	-1.1	-3.6	1.3	0.364
Australia	60	34.8	6.5	55	33.4	6.1	-1.4	-3.5	0.7	0.183
Employment Status										
Unemployed	5	38.2	11.4	5	37.0	9.7	-1.2	-16.2	13.9	0.861
Casual/Part-time/Full-time	62	34.1	6.2	56	32.8	5.9	-1.4	-3.6	0.8	0.213
Retired	31	32.8	5.5	30	31.5	5.1	-1.4	-3.9	1.2	0.301
Years with Type 2 Diabetes										
< 6 years	68	34.6	6.0	64	33.3	5.6	-1.4	-3.3	0.6	0.165
≥ 6 years or more	31	32.4	6.8	28	31.2	6.4	-1.3	-4.8	2.2	0.465
Coexisting medical conditions										
None	22	32.9	5.7	21	31.0	5.3	-1.9	-5.3	1.6	0.285
One or more	77	34.2	6.4	71	33.1	6.0	-1.1	-3.1	0.9	0.272
Activity Level										
Low	26	35.6	6.0	21	32.4	4.8	-3.3	-6.8	0.1	0.056
Medium	41	34.5	6.4	36	33.4	6.5	-1.7	-4.6	1.3	0.268
High	32	31.8	5.9	34	32.3	5.9	0.5	-2.4	3.4	0.743
Carbohydrate Intake as a %Energy kJ/day (3 months)										
Up to 26% kJ/day	75	34.5	6.3	71	33.1	6.0	-1.4	-3.4	0.6	0.175
More than 26% kJ/day	21	31.5	5.5	18	30.7	5.0	-0.9	-4.4	2.7	0.622

P values were derived from the independent sample T-test and indicate the statistical significance of the changes from baseline to 3 months of follow up. Statistical analyses were adjusted for age and gender (only in the case of total sample). SD, standard deviation from the mean; CI, confidence interval.

Table S2 *Changes in weight status and glycaemic control after 3 months*

**Weight status of participants**	**n**	**%**	**Glycaemic control**	**n**	**%**
Did not lose weight (or gained)	10	11	No change or increase in HbA1c	14	15
Lost weight	82	89	HbA1c reduction from baseline	82	85
**Changes in weight status**			**Changes in glycaemic control**		
Greater than or equal to 5% weight loss	35	38	HbA1c<6.5	50	52
Less than 5% weight loss (including weight gain)	57	62	HbA1c≥6.5	46	48

Table S3 *Adherence to Defeat Diabetes app and impact on change in HbA1c and weight loss.*

				**3-months Change**			
	**n**	**Mean**	**SD**	**Mean change**	**(95% CI) Lower**	**(95% CI) Upper**	**P value**
**Δ HbA1c (%)**				-0.5	-1.0	0.0	0.073
≤50g CHO per day	39	-1.3	1.3				
>50g CHO per day	54	-0.8	1.2				
**Weight Loss (kg)**				-2.1	-3.4	-0.7	0.003
≤50g CHO per day	39	-5.3	3.1				
>50g CHO per day	50	-3.2	3.3				
**Male**							
**Δ HbA1c (%)**				-0.4	-1.5	0.7	0.458
≤50g CHO per day	13	-1.5	1.6				
>50g CHO per day	27	-1.1	1.4				
**Weight Loss (kg)**				-1.0	-3.5	1.5	0.409
≤50g CHO per day	13	-5.1	3.6				
>50g CHO per day	25	-4.1	3.5				
**Female**							
**Δ HbA1c (%)**				-0.7	-1.2	-0.2	0.012
≤50g CHO per day	26	-1.1	1.1				
>50g CHO per day	27	-0.5	0.7				
**Weight Loss (kg)**				-3.0	-4.7	-1.4	<.001
≤50g CHO per day	26	-5.4	2.8				
>50g CHO per day	25	-2.4	3.0				

P values that compare continuous variables between genders are derived from the Independent sample T-test or the non parametric Mann-Whitney test, i.e., as per the normality of their distribution. The P values that compare categorical variables are derived from the chi-square test. Note: Small sample size is the limitation for gender comparison. CHO, Carbohydrate; SD, standard deviation from the mean; CI, confidence interval.
